# Unusual Photophysical Properties of Porphyrin-Based Supramolecular Polymers Unveiled: The Role of Metal Ligands and Side Group Amide Connectivity

**DOI:** 10.1021/acs.jpcc.3c05828

**Published:** 2023-11-21

**Authors:** Ioannis Touloupas, Elisabeth Weyandt, E. W. Meijer, Richard Hildner

**Affiliations:** † Zernike Institute for Advanced Materials, University of Groningen, Nijenborgh 4, 9747 AG Groningen, The Netherlands; ‡ Laboratory of Macromolecular and Organic Chemistry, Eindhoven University of Technology, P.O. Box 513, 5600 MB Eindhoven, The Netherlands; § Institute for Complex Molecular Systems, Eindhoven University of Technology, P.O. Box 513, 5600 MB Eindhoven, The Netherlands

## Abstract

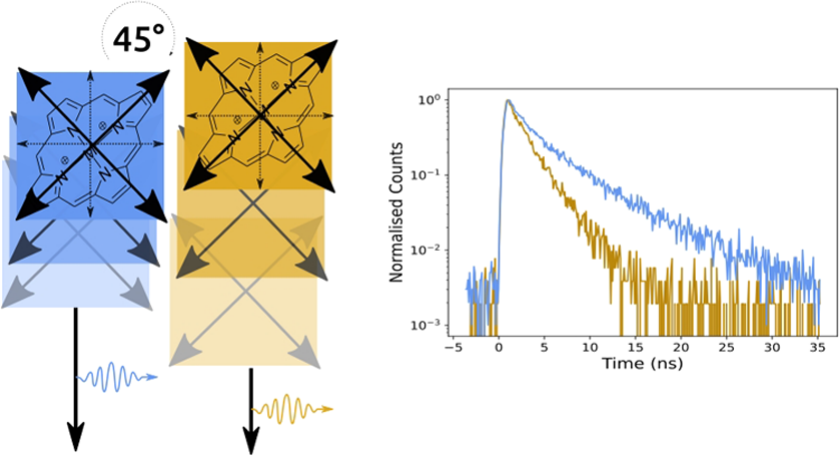

Supramolecular polymers based on porphyrins are an interesting model system, since the self-assembly and thus the photophysics can be modified by the chemical structure of the porphyrins, *e.g.*, by a metal inserted in the ligand or by different (solubilizing) side groups. Here, we investigate the photophysical properties of supramolecular polymers based on free-base and Zn-centered porphyrins, each with different amide connectivity in the side chains, by absorption and (time-resolved) photoluminescence spectroscopy on solutions. We find that for all porphyrin derivatives the B-band absorption of supramolecular polymers is a superposition of H- and J-type aggregate spectra, while the Q-band absorption indicates only J-type aggregation. The emission of supramolecular polymers stems exclusively from the Q-band and shows only J-type behavior. For supramolecular polymers based on the free-base porphyrins, we identify only a single aggregate species, whereas for Zn-centered porphyrins, two distinct species coexist in solution, each with a (slightly) different arrangement of monomers. We rationalize this complex behavior by a slip-stacking of porphyrins along the direction of one of the two B-band transition dipole moments, resulting in simultaneous H- and J-type intermolecular coupling in the B-band. In the Q-band, with its transition dipole moments oriented 45° relative to the corresponding B-band moments, only J-type coupling is thus present. Our results demonstrate that the self-assembly and the photophysics of supramolecular polymers based on porphyrins can only be fully understood if spectral information from all bands is considered.

## Introduction

Porphyrins and supramolecular assemblies of porphyrins are functional components in a variety of natural systems, such as in the photosynthetic apparatus of plants and bacteria for light harvesting and conversion into chemical energy.^1−4^ Porphyrins possess delocalized π-systems that give rise to strong absorption bands in the ultraviolet, visible, and near-infrared spectral range, depending on the precise chemical structure.^2−4^ They are therefore ideal for harvesting light over a wide spectral range. The photophysical properties are further modified in supramolecular assemblies as a result of intermolecular interactions.^5−8^ Electronic Coulomb interactions between transition dipole moments of adjacent porphyrins give rise to delocalized electronic excitations (excitons) and lead to the appearance of additional, shifted absorption bands, thus enhancing the spectral range that can be harvested. In photosynthetic reaction centers, the local dielectric protein environment around porphyrin assemblies is specifically tuned to allow for the formation of charge-separated states between molecules as the precursor for the conversion into chemical energy.^3,4,9^ Understanding the photophysics of self-assembled aggregates of porphyrins is thus not only of fundamental interest, but also can lead to a more detailed understanding of processes occurring in natural systems.

The self-assembly of porphyrins into supramolecular polymers depends on the interplay of different factors, such as monomer concentration, solvent polarity, temperature, and their chemical structure.^10−13^ The large π-system of the porphyrin core favors π-stacking or slipped stacking under suitable conditions. Further supramolecular motifs, like hydrogen-bonding amide groups in the side chains, can stabilize self-assembly into ordered supramolecular polymers. Also, a metal inserted into the ligand can have a strong impact via metal coordination.^14^ Different arrangements of porphyrins within supramolecular polymers strongly modify the photophysical properties via distinct electronic Coulomb interactions between transition dipole moments of neighboring molecules. Commonly, supramolecular polymers are classified as J-aggregates, for which the electronic interaction is negative due to a slipped stacking (or in-line arrangement) of transition dipole moments, or as H-aggregates, for which the electronic interaction is positive due to a side-by-side arrangement transition dipole moments,^15^*e.g*., for π-stacked molecules. Porphyrins show a rich and complex self-assembly behavior, and for specific porphyrins,^16^ both J-type and H-type aggregation was found, as well as an interconversion from J- to H-aggregates (or *vice versa*). Moreover, a conversion between different J- (or H-) type aggregates with only subtle differences in molecular arrangements was observed.^17−19^

The assignment of a supramolecular polymer as the J- or H-aggregate is often based on spectral shifts of UV/vis absorption spectra upon self-assembly, with a red-shift being associated with J-aggregation and a blue-shift being associated with H-aggregation. However, this approach can be ambiguous and misleading since spectral shifts are also caused by a change in the local dielectric environment of each molecule, from solvent molecules in the molecularly dissolved state to porphyrins surrounding porphyrins within a supramolecular polymer. In the latter case, the local environment is often much more polar due to the extended π-system of porphyrin cores, which leads to (red-)shifts of the transition energy of each molecule via nonresonant dispersive interactions, sometimes referred to as a gas-to-crystal shift.^8,15^ In extreme situations this latter shift can overcompensate a blue-shift due to H-aggregation, i.e., H-aggregates can feature red-shifted spectra.^20−24^ For porphyrin-based aggregates, an additional level of complexity in assignments based on shifts of optical spectra arises from the excited-state level structure with the B-band (or Soret band) in the near-UV/blue spectral range and the Q-band in the visible/near-infrared range.^25^ Each band comprises two pairwise perpendicularly oriented transition dipole moments with a 45° angle between the B- and Q-band moments.^26^ Depending on the mutual arrangement of porphyrin molecules within a supramolecular polymer, the spectral signatures from the B- and Q-band can thus be complex to interpret and can yield conflicting information, *e.g*., H-aggregation was based on B-band absorption and J-aggregation was based on spectral information from the Q-band. To alleviate this complexity, in previous studies, homochiral porphyrins have been used to study the assembly of supramolecular polymers, allowing for additional insights via the chiroptical activity of the B- and Q-bands due to the helical stacking of monomers in H-aggregates.^17−19^ However, since the B-band absorption is often substantially stronger,^17−19^ information from the Q-band is often ignored, yet required to obtain unique, unambiguous data to determine the arrangement of porphyrins within supramolecular polymers based on optical spectra.

**Figure 1:**
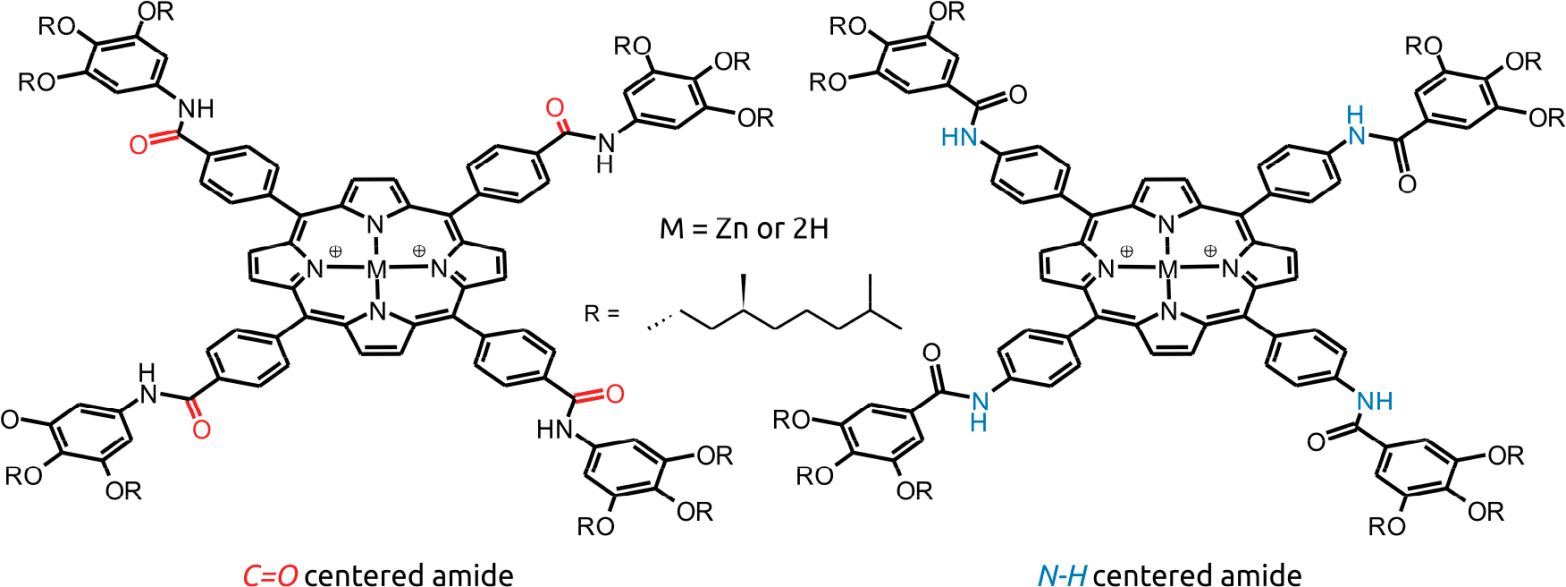
Chemical structures of the porphyrins. The C=O- (left) and N—H-centered (right) porphyrins were both studied as a free-base (2H or FB) derivative and as a Zn-centered derivative.

Here we revisit assignments of aggregate types based on porphyrin derivatives with a systematically changed chemical structure using information from both B- and Q-band absorptions, as well as (time-resolved) photoluminescence (PL) from the Q-band. Specifically, we study free-base (FB) and zinc (Zn)-centered porphyrins that each possess either C=O- or N—H-centered amides in the (*S*)-chiral side chains appended to the porphyrin core (Figure 1). We find that the complex B-band absorption of all supramolecular polymers comprises signatures of H- and J-aggregation, while the Q-band absorptions and (time-resolved) PL feature exclusively J-type signatures. This behavior can be explained by a slipped stacking of porphyrins within supramolecular polymers. Moreover, for Zn-centered porphyrins, we identified two distinct, coexisting species of supramolecular polymers in solution with a slightly different arrangement of monomers within the aggregates.

## Materials and Methods

The synthesis and supramolecular polymerization of all porphyrin derivatives (Figure 1) were reported previously.^27^ Supramolecular polymers of the porphyrins were obtained by heating the monomers in methylcyclohexane (MCH) to 90 °C, followed by ultrasonication and subsequent cooling and aging for 24 h at room temperature to assemble the monomer stacks. Samples of molecularly dissolved porphyrin monomers were prepared by dissolving the material in chloroform. The concentrations were 50 μM for all samples. UV/vis spectra were recorded on a UV 2600/2700i Shimadzu spectrometer, and steady-state photoluminescence (PL) spectra were measured with a spectrofluorimeter (LS50B, Perkin-Elmer), both in 1 cm quartz cuvettes. Time-resolved PL spectra were acquired on a home-built setup with a streak camera (C5680, Hamamatsu) in a 90° geometry by using a 2 mm quartz cuvette. The excitation source was a Ti:Sapphire laser (Mira 900, Coherent) operating at 76 MHz and tuned to a wavelength of 826 nm, which was frequency doubled to 413 nm (Model 5-050 SHG generator, INRAD) to excite into the B-band absorptions. A pulse picker (Model 9200, Coherent) was used to vary the repetition rate. A global fit^28^ of the Streak camera data was done using home-written software. The species-associated spectra were fitted using home-written python scripts. Relative PL quantum yields (PLQY), i.e., the PLQY of supramolecular polymers relative to the PLQY of the molecularly dissolved compounds, were determined as described in ref (29).

## Results and Discussion

**Figure 2:**
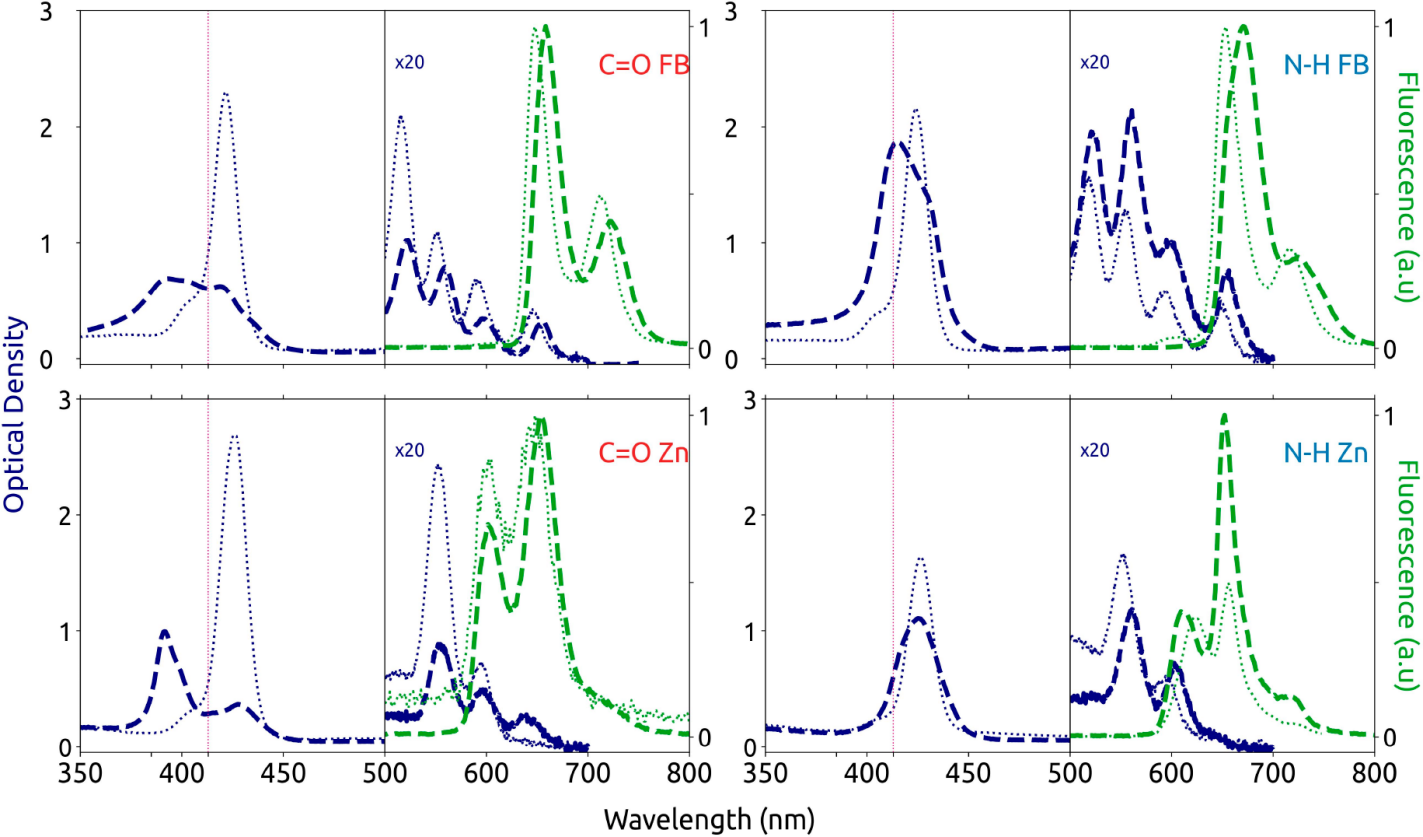
Steady-state optical spectra of the different porphyrin compounds and their supramolecular polymers. Absorption (blue) and normalized PL spectra (green) of monomers in chloroform (thin dotted) and of aggregates in MCH (thick dashed) for C=O FB (top left), N—H FB (top right), C=O Zn (bottom left), and N—H Zn (bottom right). The absorption of the Q-band is multiplied by a factor 20 for visibility. For the free-base porphyrins the Q-band absorption peaks are labeled in Figure S1 of the Supporting Information. The excitation wavelength for the acquisition of PL spectra was 413 nm and is indicated with the violet vertical lines.

In Figure 2, UV/vis absorption and steady-state photoluminescence (PL) spectra of the compounds dissolved in chloroform (molecularly dissolved monomers) as well as in MCH (supramolecular polymers) are shown. The monomers’ absorption spectra (thin dotted blue lines) feature their B-bands around 430 nm and the substantially weaker Q-bands between ∼500 and 650 nm.^14^ The B-bands are largely independent of the chemical structure and show a prominent absorption at 430 nm and a shoulder at around 405 nm, corresponding to the 0–0 and 0–1 transitions of the degenerate B_*x*_- and B_*y*_-bands. The Q-bands of the Zn-centered porphyrins (C=O Zn, N—H Zn, bottom row) feature a single vibronic progression of degenerate Q_*x*_- and Q_*y*_-bands with the 0–0 transition at ∼600 nm and the 0–1 transition at ∼550 nm. In contrast, the reduced symmetry in free-base porphyrins (C=O FB, N—H FB, top row) lifts degeneracy in the Q-band and two spectrally shifted vibronic progressions are visible (see SI, Figure S1, for the assignment of peaks).

The PL of all molecularly dissolved compounds (Figure 2, dotted green) stems exclusively from the (lowest-energy/longest-wavelength) Q-band due to rapid internal conversion prior to the emission process.^14^ The spectral shift between the longest-wavelength absorption and the 0–0 PL peaks is always around 300 cm^–1^. For the free-base porphyrins, the electronic 0–0 PL peak around 660 nm is more intense than the 0–1 PL peak at 710 nm, while for the Zn-centered porphyrins the 0–0 transition appears at ∼620 nm and is weaker compared to the 0–1 peak at 650 nm. Time-resolved PL measurements yield a monoexponential decay with an excited state lifetime of ∼4.2 ns for all compounds molecularly dissolved in chloroform (Table 1).

**Table 1:** Excited-State Lifetimes of the Different Porphyrin Compounds, Molecularly Dissolved in Chloroform (Monomer), and of the Emitting Aggregate Species in MCH

	lifetime (ns)		
compound	monomer	J_1_	J_2_
C=O FB	4.1	10.3	
C=O Zn	4.2	6.2	1.9
N—H FB	4.2	10.8	
N—H Zn	4.2	7.2	2.1

The absorption spectra of supramolecular polymers in MCH are shown in Figure 2 as thick dashed blue lines. Compared with the monomer absorptions, the B-bands of supramolecular polymers feature a strong change in their spectral shape. The shortest-wavelength absorption at 380 nm appears to gain oscillator strength at the expense of that at 430 nm, indicating H-type aggregation.^30^ We note, however, that the complex spectral shapes leave some ambiguity in interpretation and coexisting J- (H-) aggregates have been suggested, too.^17^ The Q-band absorptions of supramolecular polymers are, in general, slightly red-shifted by ∼ 250 cm^–1^ with some change of the spectral shape compared to the monomer absorptions. For the free-base porphyrin aggregates the intensities of the 0–0 peaks, relative to those of the 0–1 peaks, of both the Q_*x*_- and Q_*y*_-bands are slightly more intense compared to the monomer absorptions (SI, Figure S1). For Zn-centered porphyrins, an additional longer-wavelength (lower-energy) peak around 650 nm emerges upon supramolecular polymerization. These spectral changes in the Q-bands are characteristic for J-type aggregation^15^ with a slipped stacking of Q-band transition dipole moments, whereas the changes in the B-bands rather indicate H-aggregation with the B-band transition dipole moments arranged side-by-side, yielding rich, but complex, photophysics.

The steady-state PL spectra of supramolecular polymers are shown in Figure 2 as thick dashed green lines. As for the monomer PL, here we exclusively observe emission from the longest-wavelength (lowest-energy) Q-band as well; i.e., the PL spectra reflect only the arrangement of Q-band transition dipole moments within aggregates. For C=O FB and N—H FB based aggregates (top row) the 0–0 and 0–1 PL peaks appear at around 670 and 720 nm, respectively, both with a small red-shift of ∼300 cm^–1^ compared to the monomer PL. Since the relative intensity of the 0–0 PL peak of the supramolecular polymers is slightly higher compared to that of the corresponding monomer PL, this suggests that the Q-band emission stems from J-aggregates. The PL spectra of supramolecular polymers based on Zn-centered porphyrins possess three peaks (bottom row), and the lowest-wavelength peak at ∼620 nm is always weaker in relative intensity compared to the most intense peak at 670 nm. Moreover, the PL spectra strongly overlap with the Q-band absorption in the range 600–650 nm, which renders an unambiguous assignment of peaks difficult. Together with the complex spectral shapes of the absorptions of supramolecular polymers, this indicates that especially Zn-centered porphyrins feature a complex self-assembly behavior in MCH with coexisting aggregate species.

**Figure 3:**
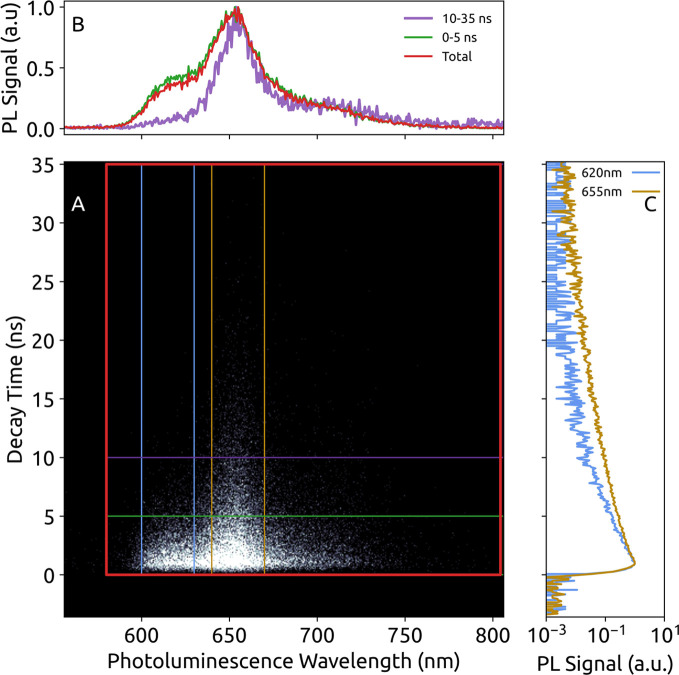
Time-resolved PL spectroscopy of supramolecular polymers based on N—H Zn in MCH solution. The PL intensity of the Streak image (A) is given as gray scale, and the colored boxes indicate time- and wavelength-intervals, from which transient PL spectra (B) and PL decay curves (C) have been extracted. In C the legends label the center wavelengths, the total wavelength interval is 30 nm each. The PL stems exclusively from the Q-band that is populated by internal conversion upon excitation into the B-band at a wavelength of 413 nm.

To gain further insight into the self-assembly behavior of the porphyrin derivatives, we performed time-resolved PL spectroscopy using a Streak camera in combination with a global fitting analysis of the data (see Materials and Methods). This approach allows distinguishing different aggregate species based on their Q-band emission via different excited-state lifetimes, even if their PL spectra overlap. In Figure 3A we show the Streak data of supramolecular polymers of N—H Zn in MCH upon excitation at a wavelength of 413 nm as an example and for illustration of data analysis. The horizontal axis in the Streak data represents detection wavelength, the vertical axis is time after the excitation pulse, and the PL signal is displayed in gray scale. Transient PL spectra are retrieved by integrating over two intervals along the time axis (0–5 ns and 10–35 ns, Figure 3B, green and violet). The transient spectra are overlaid with the time-integrated PL spectrum (Figure 3B top, red), which is identical with the steady-state PL (Figure 2). In the transient spectra particularly the PL signal around 620 nm is strongly time-dependent (see the green and violet spectra). PL decay curves, spectrally integrated over an interval of 30 nm around a central wavelength of 620 and 655 nm, are shown in Figure 3C. Both PL decay curves are nonexponential with the overall decay being faster in the short-wavelength interval (brown) and slower in the long-wavelength interval (blue).

The visual inspection of Streak data of N—H Zn-based aggregates (Figure 3) leaves several options for interpretation: (i) (at least) two different emitting aggregate species with distinct lifetimes are present; (ii) one aggregate species and the remaining molecularly dissolved monomer are present; or (iii) one aggregate species is present and energy transfer from higher-energy to lower-energy states within each individual aggregate takes place. We can rule out a monomer contribution to the PL signal (option (ii)), since the lifetimes of N—H Zn-based aggregates determined from the Streak data are different from the monomer lifetime (Table 1). Energy transfer from higher- to lower-energy states [option (iii)] can be excluded, too, since we do not observe a rising component in the decay curve at long detection wavelength (low energy) that matches the faster decay at short wavelength (high energy). The rise of the decay curves is determined by the instrument response function for all detection wavelengths. Hence, only option (i) remains, the coexistence of different emitting aggregate species based on N-H Zn that emit independently from each other and do not “communicate” via energy transfer. The Streak camera data for supramolecular polymers of C=O Zn porphyrins show a similar behavior with nonexponential and wavelength-dependent PL decays, thus, indicating the coexistence of (at least) two emitting species as well (SI, Figure S2). We note that two coexisting emitting aggregate species for Zn-centered porphyrins, as found here from the Q-band emission, imply the presence of two distinct H- and J-aggregates based on the B-band absorption (see the discussion further below). The Streak data of supramolecular polymers of the free-base porphyrins (SI, Figures S3 and S4) feature monoexponential PL decays that do not change as a function of the detection wavelength, and the transient spectra do not change as a function of time. These data thus imply the presence of only one emitting aggregate species for free-base porphyrin supramolecular polymers (and, by extension, one aggregate species based on B-band absorption).

**Figure 4:**
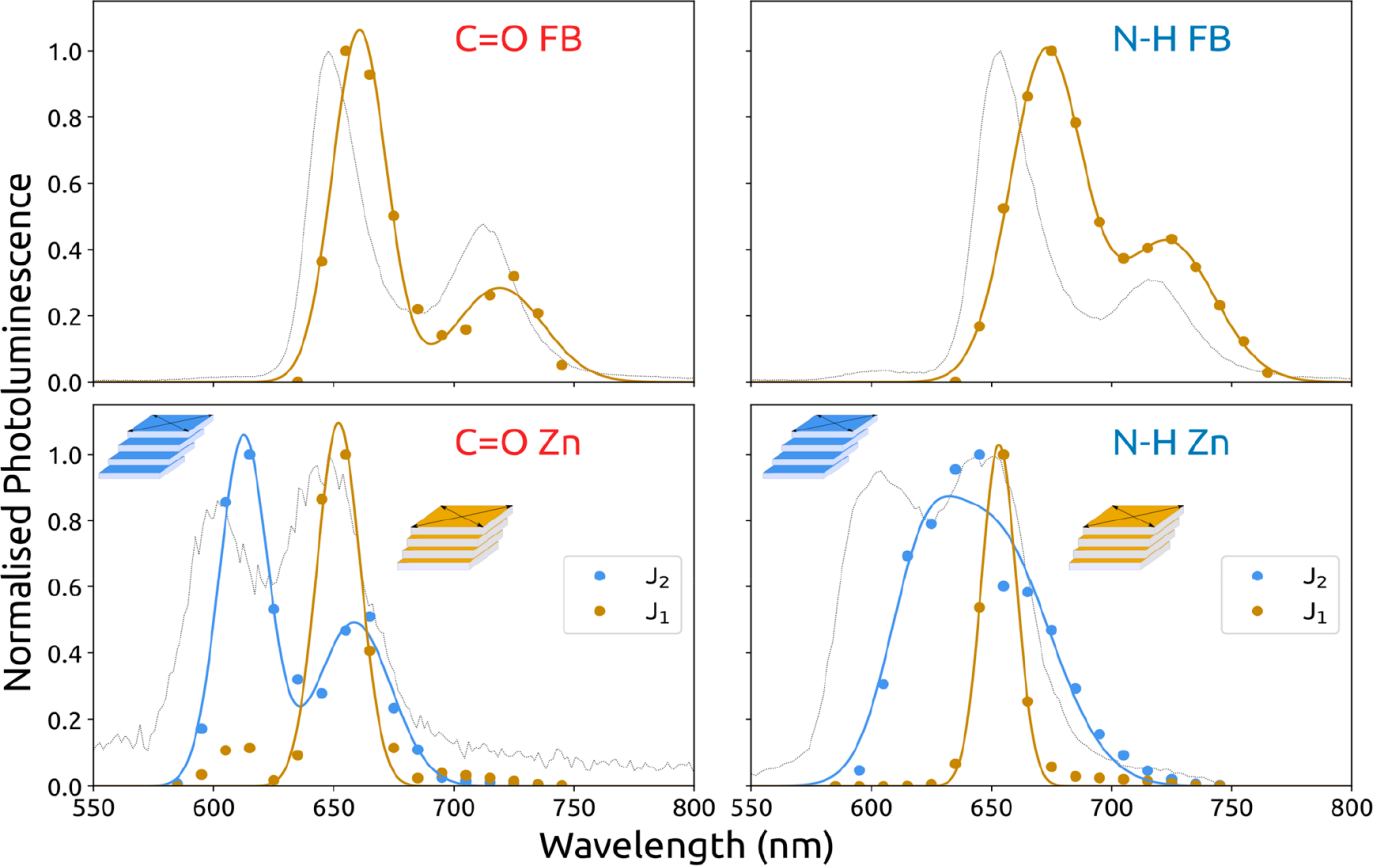
Species-associated spectra derived from time-resolved PL spectroscopy on the Q-bands of supramolecular polymers. The colored filled dots indicate the species-associated spectra for the emitting aggregate species. For Zn-centered porphyrins brown dots label the spectra of the J_1_-species, and the blue dots indicate spectra of the J_2_-species. The solid colored lines are fits to the species-associated spectra, and the thin gray lines show the PL spectra of the corresponding monomers in chloroform for comparison. The insets illustrate the different stacking of Zn-centered porphyrins into the coexisting aggregate species (J_1_: brown, J_2_: blue).

To obtain information on the nature of the (coexisting) emitting aggregate species, we employ a global fitting analysis. The Streak data are integrated over intervals of 10 nm along the wavelength axis, and each retrieved PL decay curve is fitted by a (multi)exponential function including deconvolution of the instrument response function. For the optimum global fit, the lifetimes are constant as a function of the detection wavelength, and only the relative amplitudes of the exponentials vary. Plotting the amplitudes as a function of wavelength, we obtain species-associated spectra that are presented in Figure 4 (filled dots) together with fits to the spectra (solid colored lines). For comparison, we overlay in Figure 4 the corresponding steady-state PL spectra of the molecularly dissolved compounds as gray solid lines. The lifetime components corresponding to the species-associated spectra are given in Table 1. We recorded time-resolved PL spectra using a shorter excitation wavelength of 389 nm (see SI, section 4), where exclusively supramolecular polymers are excited (see Figure 2). The results are consistent with those using 413 nm excitation. We also recorded time-resolved PL spectra as a function of laser repetition rate and fluence of the excitation pulses as well. Within the range of accessible repetition rates and fluences the lifetimes and species-associated spectra do not change (see SI, Figures S5–S8). Hence, nonlinear effects, such as exciton–exciton annihilation, do not impact our time-resolved data.

For the supramolecular polymers based on free-base porphyrins, a single species-associated spectrum is found (Figure 4, top row) that we ascribe to J-type emission from the Q-band, i.e., the transition dipole moments in the Q-band must be arranged in a slip-stacked fashion (but not necessarily those in the B-band, see further below). The species-associated spectra feature a 0–0 PL peak at 670 nm and a 0–1 PL peak at 720 nm and agree well with the corresponding steady-state PL spectra of the supramolecular polymers in Figure 2 (top row). The spectral shift between the longest-wavelength (lowest-energy) absorption and the 0–0 PL peak is small at ∼120 cm^–1^ (C=O FB) and ∼250 cm^–1^ (N—H FB). The relative 0–0 PL peak intensity is slightly higher (C=O FB) or roughly equal (N—H FB) compared to the peak intensity ratio of the monomer PL. Finally, the line width of the 0–0 PL peaks is reduced compared to that of the monomers (Table 2). All these spectral features are characteristic for J-type emission.^15^ The lifetimes corresponding to the species-associated spectra are 10.3 ns (C=O FB) and 10.8 ns (N—H FB), respectively, and are thus longer than the monomer lifetimes (Table 1). This discrepancy can be rationalized by a reduced nonradiative decay rate upon aggregation, *e.g*., by freezing-out of high-energy vibrations that promote internal conversion or by planarization of the porphyrin core. Hence, the total decay rate decreases (the measured lifetime increases) for the supramolecular polymers; see SI, section S3.

**Table 2:** Linewidths of the Highest-Energy (Lowest-Wavelength) PL Peaks for the Different Porphyrin Compounds as Monomer, Molecularly Dissolved in Chloroform, and for the Emitting J-Aggregate Species in MCH^a^.

	linewidth (cm^–1^)			
compound	monomer	J_1_	J_2_	CD
C=O FB	396	240		strong
C=O Zn	368	208	262	strong
N—H FB	394	330		weak
N—H Zn	471	168	370	none

a For the aggregate species, the linewidths were extracted from the species-associated spectra. The relative strength of the circular-dichroism (CD) signal from the B-band of supramolecular polymers has been taken from refs (10 and 17).

Supramolecular polymers based on Zn-centered porphyrins feature two J-type emitting species (Figure 4, bottom row). We label those aggregates as J_1_ emitting at longer wavelengths (lower energy) and J_2_ emitting at shorter wavelengths (higher energy). The species-associated spectra of the J_2_-aggregates (at shorter wavelengths, blue) feature a 0–0 PL peak around 610 nm and a 0–1 PL peak at around 660 nm. The relative 0–0 PL intensity is higher, and the 0–0 line width is narrower for the J_2_-aggregate as compared to the monomer PL (Figure 4, gray line, and Table 2). Moreover, the shift between the 0–0 PL at around 610 nm and the aggregate absorption peak at around 600 nm is only about 170 cm^–1^. Finally, the lifetimes of this species are shorter than that of the monomers (Table 1), which all are consistent with J-type aggregation of this emitting J_2_-species. For the J_1_-aggregates of Zn-centered porphyrins (at longer wavelengths, brown), we only resolve a single peak in the species-associated spectra (probably due to the overall low PL signal), leaving some ambiguity in the assignment. However, the species-associated spectra exhibit very narrow linewidths of 208 cm^–1^ (C=O Zn) and 168 cm^–1^ (N—H Zn). In fact, those are the narrowest lines of all supramolecular polymers being a factor of 2–3 narrower than the 0–0 PL peaks in the monomer spectra (Table 2). The species-associated spectra of the J_1_-aggregates possess a spectral shift of 310 cm^–1^ (C=O Zn) and 270 cm^–1^ (N—H Zn) relative to the additional absorption peak that appears around 650 nm upon supramolecular polymerization of the Zn-centered porphyrins. Those data strongly indicate J-type emission, too, with a slip-stacked arrangement of Q-band transition dipole moments. The longer lifetimes of the J_1_-aggregates compared to the monomers’ lifetimes (Table 1) can again be understood by a reduction in nonradiative decay rates upon aggregation, see above and SI, section S3.

**Figure 5:**
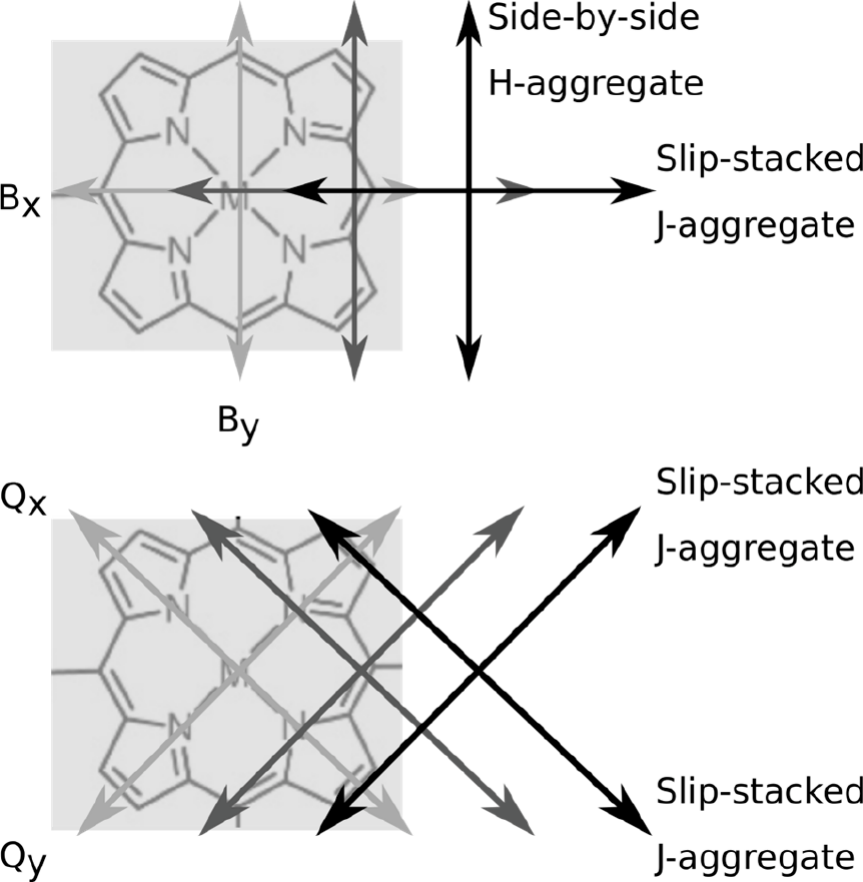
Illustration of the arrangements of transition dipole moments in supramolecular polymers based on porphyrins. Top: B-band transition dipole moments. Bottom: Q-band transition dipole moments. The gray scale of the arrows indicate the position within the stack (light gray: bottom; black: top).

Our observations of spectral signatures of (coexisting) emitting J-type species and the complex B-band absorptions of the supramolecular polymers based on the different porphyrin compounds highlight their intricate self-assembly behavior in MCH. To start with, we reiterate that the PL spectra of all supramolecular polymers stem exclusively from the Q-band and show exclusively the presence of emitting J-aggregates. The clear differences in lifetimes (Table 1) and shapes of the species-associated spectra of supramolecular polymers (Figure 4) as compared to lifetimes and spectra of monomers demonstrate that monomer signals are not detectable. Moreover, null- or X-type aggregates were not present here. In such aggregates the monomers are arranged such that the electronic interactions within the aggregate cancel, which would leave lifetime and spectra unchanged.^31,32^ The porphyrin monomers must therefore be arranged such that the Q-band transition dipole moments are slip-stacked.^6^ This slipped stacking is in line with the J-type features observed in the Q-band absorptions (Figures 2 and S1). The B-band absorptions, in contrast, rather indicate H-type aggregation, usually assumed to result from cofacial stacking with a side-by-side arrangement of B-band transition dipole moments (although this assignment can be ambiguous; see Figure 2). This discrepancy can be resolved, considering that in both the B- and Q-band of porphyrins the transition dipole moments are pairwise perpendicularly oriented: The B_*x*_- and B_*y*_-transition dipole moments are oriented along the axes connecting opposite *meso*-positions, and the axes of the Q_*x*_- and Q_*y*_-transition dipole moments connect opposite pyrrole units,^33^ i.e., the latter are rotated by 45° relative to the corresponding B-band components (Figure 5). Recent work^10,17^ suggests that in J-aggregates of the porphyrins studied here, the slip is along the direction of a B-band transition dipole moment. Assuming a slip, for example, along the direction of the B_*x*_-component, the B_*x*_-band represents a J-aggregate due to the slip-stacked arrangement of B_*x*_-transition dipole moments of adjacent molecules. The corresponding B_*y*_-components, however, are still side-by-side with respect to each other, and thus, the B_*y*_-band response is that of an H-aggregate (Figure 5, top). Although for a given slip the magnitude of the Coulomb interactions between the B_*x*_-transition dipole moments (J-type) are roughly by a factor of 2 stronger than those between the B_*y*_-components (H-type),^33^ both signatures are visible and give rise to the complex spectral shapes that we observe in the B-band absorptions of the supramolecular polymers (Figure 2). For the Q-band, the slip along the B_*x*_-direction translates into a slip-stacked arrangement of both the Q_*x*_- and Q_*y*_-components (with a lateral offset, see Figure 5, bottom). Hence, the Q-bands exhibit exclusively J-type features in both absorption and PL spectra, as we indeed observe (see Figures 2, 4, and S1). In other words, the assignment of aggregation (H- and J-type) depends on the band (B and Q) that is observed, and the full picture can only be retrieved if information from all bands is included.

Importantly, the slip along the direction of a B-band transition dipole moment and, thus, the slip of the Q-band moments, is not necessarily as large as commonly assumed for the slipped stacking in J-aggregates. Typically, a slip ≥0.5 nm is required to switch from H- to J-type behavior according to the classical Kasha model, where only Coulomb interactions are considered.^34^ However, a 0.5 nm slip is too large to allow for hydrogen bonds to form in particular in chiral C=O connected porphyrins.^17^ In stacked arrangements of molecules, as present in our supramolecular polymers, the ground and excited-state wave functions of neighboring molecules can overlap, and charge-transfer mediated (CT) interactions (superexchange interactions) can play an important role. In fact, those CT interactions can dominate over Coulomb interactions between transition dipole moments and thus dominate the photophysics.^34−36^ In analogy to the classical Kasha model of aggregates, a positive CT interaction results in H-aggregates and a negative one gives rise to J-aggregates. The sign of CT interactions is determined by the sign of the electron and hole transfer integrals, which in turn are determined by the nodal patterns of the relevant ground and excited-state wave functions. Since those nodal patterns modulate very rapidly within a molecule, a rather small (sub-)Ångstrom slip can be sufficient to “switch” from positive (H-type) to negative interactions (J-type) for the slip-stacked component in the B-band and for both Q-band moments. This is known as unconventional (non-Kasha) aggregation and has been observed for several aggregates based on small molecules.^35^ To clarify the nature of interactions in our porphyrin-based supramolecular polymers, however, detailed and complex quantum-chemical calculations are required,^37^ which are beyond the scope of this work.

The (inhomogeneous) linewidths of the (J-type) emitting species (Table 2) allow us to draw conclusions about (electronic, structural) order/disorder of the supramolecular polymers formed in MCH. Electronic disorder is caused by a heterogeneous dielectric environment around the porphyrin cores (e.g., determined by specific arrangements of or within the side chains) and randomly shift the transition energies of monomers within an aggregate; structural disorder results from a nonperfect mutual arrangement of monomers and yields variations of electronic Coulomb interactions between transition dipole moments (or of CT interactions).^6,8^ This disorder can vary along a single aggregate (intraaggregate disorder) and between aggregates (interaggregate disorder) or can be a combination of both. Although we cannot distinguish electronic and structural as well as intra- and interaggregate disorder, we are nevertheless able to gain useful insights from the measured linewidths. Generally, we find that supramolecular polymers based on C=O-centered porphyrins (with the exception of the J_1_-species of N—H Zn) show the narrowest lines and are thus most ordered, implying delocalized excitons along an aggregate. Previous calculations on the amide connectivity in free-base compounds found that the C=O connected side groups are rotated out of the porphyrin plane.^17^ This results in a lower interaction energy for the association of monomers to an aggregate, i.e., more stable aggregates and, as our data suggest (electronically and structurally), more ordered aggregates form. Comparing aggregates based on free-base porphyrins with the J_1_-species of Zn-centered porphyrins, the latter appear more ordered. Metal coordination contributes an additional interaction (next to hydrogen-bonding and π-stacking) that stabilizes the assembly into supramolecular polymers. The J_2_-species in Zn-centered porphyrin aggregates is (significantly) less ordered, thus excitons are more localized, compared to the J_1_-species. The PL of the J_2_-species is blue-shifted with respect to that of the J_1_-aggregates (Figure 4), but only slightly shifted relative to the monomer PL around 600 nm (Figure 2, bottom). This indicates a weaker interaction between Q-band transition dipole moments for the J_2_-species, such that the exciton bandwidth is smaller, and spectral shifts relative to the monomer PL (and absorptions) are also smaller. Weaker interactions result from a slightly different arrangement of monomers within this second aggregate species, probably from a slightly larger slip along a B-band component (see the sketches in Figure 4, bottom row). This second coexisting aggregate species represents the minority species and contributes only about 20% to the total PL signal (for both Zn-centered porphyrins). Since the absolute PL quantum yields are unknown, this value cannot be directly translated into an absolute abundance of this second species. It nevertheless indicates that this second coexisting aggregate species is less favorably formed in MCH, but once formed it is stable.

Finally, we comment on the chiral features of the supramolecular polymers observed in circular dichroism (CD) spectra from the B-band of the porphyrin compounds with their (*S*)-chiral groups in the side chains studied here. Supramolecular polymers based on the C=O-centered porphyrins feature a pronounced Cotton effect, whereas N—H FB-based aggregates feature only weak CD signals, and in N—H Zn-based supramolecular polymers, those are absent (Table 2).^10,17,27^ Previously, this behavior was attributed to the formation of cofacially stacked, chiral H-aggregates (strong CD signal) or the formation of disordered, achiral J-aggregates with a pronounced slipped stacking so that the amides in the side groups cannot form hydrogen bonds and cannot enforce a chiral assembly (weak/no CD signal). However, we have shown here that all supramolecular polymers feature a slipped stacking; the slip can be small in the case of unconventional (non-Kasha) aggregation, and hydrogen bonding can thus still stabilize self-assembly into chiral aggregates for all compounds. Comparing the appearance of CD signals and the (electronic/structural) order of the aggregates formed, as judged from the PL linewidths (see Table 2), there is a clear correlation between higher order and the presence of a pronounced CD signal (with the exception of the second species of N—H Zn-based aggregates). CD spectroscopy on chiral assemblies of electronically interacting molecules is known to be very sensitive to non-nearest-neighbor interactions and exciton delocalization.^33^ Thus, strong CD signals require (at least locally) ordered aggregates that support delocalized excitons, consistent with our data. For N—H Zn-based supramolecular polymers, we can only speculate about the absence of CD signals in the B-band. The rather broad, unstructured B-band absorption, representing a superposition of the absorption of both species, might not allow the detection of the CD signal from the more ordered aggregate species of this compound (*e.g*., due to a cancellation by two spectrally shifted Cotton effects).

## Conclusion

We studied supramolecular polymers based on free-base and Zn-centered porphyrins by (time-resolved) optical spectroscopy. All supramolecular polymers have in common that the porphyrins are assembled in a stacked arrangement yet with a (small) slip along one of the components of the B-band transition dipole moments. Hence, the B-band absorption shows a superposition of H- and J-type aggregation, while the Q-band absorption and emission show exclusively J-type spectra. Particularly Zn-centered porphyrins feature a complex self-assembly behavior and form two distinct, coexisting aggregate species with (slightly) different arrangement of monomers within supramolecular polymers, which is manifest in distinct excited-state lifetimes of the Q-band PL. In the case of a small slip stack in all aggregates, a helical stacking of the monomers, stabilized by hydrogen-bonding amides in the chiral side groups, is still possible. This naturally can lead to helical structures that have been observed for supramolecular polymers based on Zn-centered porphyrins. Since the J-type PL is highly sensitive to (electronic and structural) order, this allowed us to establish that C=O-centered porphyrins form more ordered aggregates than their N-H centered counterparts due to the smaller interaction energy for the association in the case of C=O-connected amides. More ordered aggregates imply a stronger exciton delocalization along a supramolecular polymer, which is beneficial for energy transport, and thus for potential applications. Our time-resolved PL measurements on supramolecular polymers based on porphyrin derivatives allowed us to elucidate the nature of the aggregates, the degree of order within the aggregates, as well as the coexistence of aggregate species for Zn-centered porphyrins, which was not possible by considering only absorption data.

## Supporting Information Available

Expanded Q-band absorption of free-base porphyrins, additional streak camera and lifetime data, nonradiative decay rates and relative PLQY, and Streak data upon excitation at 389 nm (PDF)

## References

[ref1] Jordan P.; Fromme P.; Witt H. T.; Klukas O.; Saenger W.; Krauß N. Three-Dimensional Structure of Cyanobacterial Photosystem I at 2.5 Å Resolution. Nature 2001, 411 (6840), 909–917. 10.1038/35082000. 11418848

[ref2] Miyatake T.; Tamiaki H. Self-Aggregates of Bacteriochlorophylls-c, d and e in a Light-Harvesting Antenna System of Green Photosynthetic Bacteria: Effect of Stereochemistry at the Chiral 3-(1-Hydroxyethyl) Group on the Supramolecular Arrangement of Chlorophyllous Pigments. Journal of Photochemistry and Photobiology C: Photochemistry Reviews 2005, 6 (2), 89–107. 10.1016/j.jphotochemrev.2005.06.001.

[ref3] Scholes G. D.; Fleming G. R.; Olaya-Castro A.; van Grondelle R. Lessons from Nature about Solar Light Harvesting. Nature Chem 2011, 3 (10), 763–774. 10.1038/nchem.1145. 21941248

[ref4] Croce R.; van Amerongen H. Natural Strategies for Photosynthetic Light Harvesting. Nat Chem Biol 2014, 10 (7), 492–501. 10.1038/nchembio.1555. 24937067

[ref5] Keijer T.; Bouwens T.; Hessels J.; Reek J. N. H. Supramolecular Strategies in Artificial Photosynthesis. Chemical Science 2021, 12 (1), 50–70. 10.1039/D0SC03715J. PMC8179670 34168739

[ref6] Brixner T.; Hildner R.; Köhler J.; Lambert C.; Würthner F. Exciton Transport in Molecular Aggregates - From Natural Antennas to Synthetic Chromophore Systems. Advanced Energy Materials 2017, 7 (16), 1700236 10.1002/aenm.201700236.

[ref7] Drain C. M.; Varotto A.; Radivojevic I. Self-Organized Porphyrinic Materials. Chemical Reviews 2009, 109 (5), 1630–1658. 10.1021/cr8002483. 19253946 PMC2681784

[ref8] Kreger K.; Schmidt H.-W.; Hildner R. Tailoring the Excited-State Energy Landscape in Supramolecular Nanostructures. Electron. Struct. 2021, 3 (2), 023001 10.1088/2516-1075/abf485.

[ref9] McHale J. L. Hierarchal structure of light-harvesting porphyrin aggregates | J-Aggregates. J-Aggregates; World Scientific, 2012; Vol. 2, pp 77–118.

[ref10] Mabesoone M. F. J.; Markvoort A. J.; Banno M.; Yamaguchi T.; Helmich F.; Naito Y.; Yashima E.; Palmans A. R. A.; Meijer E. W. Competing Interactions in Hierarchical Porphyrin Self-Assembly Introduce Robustness in Pathway Complexity. J. Am. Chem. Soc. 2018, 140 (25), 7810–7819. 10.1021/jacs.8b02388. 29886728 PMC6026832

[ref11] van der Weegen R.; Teunissen A. J. P.; Meijer E. W. Directing the Self-Assembly Behaviour of Porphyrin-Based Supramolecular Systems. Chem.–Eur. J. 2017, 23 (15), 3773–3783. 10.1002/chem.201605872. 28111823

[ref12] Wan Y.; Stradomska A.; Knoester J.; Huang L. Direct Imaging of Exciton Transport in Tubular Porphyrin Aggregates by Ultrafast Microscopy. J. Am. Chem. Soc. 2017, 139 (21), 7287–7293. 10.1021/jacs.7b01550. 28480703

[ref13] Kim T.; Ham S.; Lee S. H.; Hong Y.; Kim D. Enhancement of Exciton Transport in Porphyrin Aggregate Nanostructures by Controlling the Hierarchical Self-Assembly. Nanoscale 2018, 10 (35), 16438–16446. 10.1039/C8NR05016C. 30141821

[ref14] Gouterman M. Spectra of Porphyrins. J. Mol. Spectrosc. 1961, 6 (C), 138–163. 10.1016/0022-2852(61)90236-3.

[ref15] Spano F. C. The Spectral Signatures of Frenkel Polarons in H- and J-Aggregates. Acc. Chem. Res. 2010, 43 (3), 429–439. 10.1021/ar900233v. 20014774

[ref16] Maiti N. C.; Mazumdar S.; Periasamy N. J- and H-Aggregates of Porphyrin-Surfactant Complexes: Time-Resolved Fluorescence and Other Spectroscopic Studies. J. Phys. Chem. B 1998, 102 (9), 1528–1538. 10.1021/jp9723372.

[ref17] Weyandt E.; Filot I. A. W.; Vantomme G.; Meijer E. W. Consequences of Amide Connectivity in the Supramolecular Polymerization of Porphyrins: Spectroscopic Observations Rationalized by Theoretical Modelling. Chem.–Eur. J. 2021, 27 (37), 9700–9707. 10.1002/chem.202101036. 33938050 PMC8362183

[ref18] Weyandt E.; Leanza L.; Capelli R.; Pavan G. M.; Vantomme G.; Meijer E. W. Controlling the Length of Porphyrin Supramolecular Polymers via Coupled Equilibria and Dilution-Induced Supramolecular Polymerization. Nature Communications 2022, 13 (1), 248 10.1038/s41467-021-27831-2. PMC8752679 35017511

[ref19] Weyandt E.; Mabesoone M. F. J.; de Windt L. N. J.; Meijer E. W.; Palmans A. R. A.; Vantomme G. How to Determine the Role of an Additive on the Length of Supramolecular Polymers?. Organic Materials 2020, 02 (02), 129–142. 10.1055/s-0040-1708813.

[ref20] Wittmann B.; Wenzel F. A.; Wiesneth S.; Haedler A. T.; Drechsler M.; Kreger K.; Köhler J.; Meijer E. W.; Schmidt H.-W.; Hildner R. Enhancing Long-Range Energy Transport in Supramolecular Architectures by Tailoring Coherence Properties. J. Am. Chem. Soc. 2020, 142 (18), 8323–8330. 10.1021/jacs.0c01392. 32279503 PMC7212519

[ref21] Stäter S.; Wenzel F. A.; Welz H.; Kreger K.; Köhler J.; Schmidt H.-W.; Hildner R. Directed Gradients in the Excited-State Energy Landscape of Poly(3-Hexylthiophene) Nanofibers. J. Am. Chem. Soc. 2023, 145 (25), 13780–13787. 10.1021/jacs.3c02117. 37315116 PMC10311527

[ref22] Spano F. C. Modeling Disorder in Polymer Aggregates: The Optical Spectroscopy of Regioregular Poly(3-Hexylthiophene) Thin Films. The Journal of Chemical Physics 2005, 122 (23), 234701 10.1063/1.1914768. 16008467

[ref23] Clark J.; Chang J.-F.; Spano F. C.; Friend R. H.; Silva C. Determining Exciton Bandwidth and Film Microstructure in Polythiophene Films Using Linear Absorption Spectroscopy. Appl. Phys. Lett. 2009, 94 (16), 163306 10.1063/1.3110904.

[ref24] Raithel D.; Baderschneider S.; de Queiroz T. B.; Lohwasser R.; Köhler J.; Thelakkat M.; Kümmel S.; Hildner R. Emitting Species of Poly(3-Hexylthiophene): From Single, Isolated Chains to Bulk. Macromolecules 2016, 49 (24), 9553–9560. 10.1021/acs.macromol.6b02077.

[ref25] Marek P. L.; Hahn H.; Balaban T. S. On the Way to Biomimetic Dye Aggregate Solar Cells. Energy Environ. Sci. 2011, 4 (7), 2366–2378. 10.1039/c1ee01053k.

[ref26] Prendergast K.; Spiro T. G. Predicted Geometries of Porphyrin Excited States and Radical Cations and Anions. J. Phys. Chem. 1991, 95 (24), 9728–9736. 10.1021/j100177a025.

[ref27] Helmich F.; Lee C. C.; Nieuwenhuizen M. M. L.; Gielen J. C.; Christianen P. C. M.; Larsen A.; Fytas G.; Leclère P. E. L. G.; Schenning A. P. H. J.; Meijer E. W. Dilution-Induced Self-Assembly of Porphyrin Aggregates: A Consequence of Coupled Equilibria. Angewandte Chemie International Edition 2010, 49 (23), 3939–3942. 10.1002/anie.201000162. 20379987

[ref28] van Stokkum I. H. M.; Larsen D. S.; van Grondelle R. Global and Target Analysis of Time-Resolved Spectra. Biochimica et Biophysica Acta (BBA) - Bioenergetics 2004, 1657 (2–3), 82–104. 10.1016/j.bbabio.2004.04.011. 15238266

[ref29] Brouwer A. M. Standards for Photoluminescence Quantum Yield Measurements in Solution (IUPAC Technical Report). Pure Appl. Chem. 2011, 83 (12), 2213–2228. 10.1351/PAC-REP-10-09-31.

[ref30] Spano F. C.; Silva C. H- and J-Aggregate Behavior in Polymeric Semiconductors. Annu. Rev. Phys. Chem. 2014, 65, 477–500. 10.1146/annurev-physchem-040513-103639. 24423378

[ref31] Hestand N. J.; Spano F. C. Interference between Coulombic and CT-Mediated Couplings in Molecular Aggregates: H- to J-Aggregate Transformation in Perylene-Based π-Stacks. The Journal of Chemical Physics 2015, 143 (24), 244707 10.1063/1.4938012. 26723702

[ref32] Gierschner J.; Shi J.; Milián-Medina B.; Roca-Sanjuán D.; Varghese S.; Park S. Luminescence in Crystalline Organic Materials: From Molecules to Molecular Solids. Advanced Optical Materials 2021, 9 (13), 2002251 10.1002/adom.202002251.

[ref33] Satake A.; Kobuke Y. Artificial Photosynthetic Systems: Assemblies of Slipped Cofacial Porphyrins and Phthalocyanines Showing Strong Electronic Coupling. Org. Biomol. Chem. 2007, 5 (11), 1679–1691. 10.1039/b703405a. 17520134

[ref34] Hestand N. J.; Spano F. C. Expanded Theory of H- and J-Molecular Aggregates: The Effects of Vibronic Coupling and Intermolecular Charge Transfer. Chem. Rev. 2018, 118 (15), 7069–7163. 10.1021/acs.chemrev.7b00581. 29664617

[ref35] Spano F. C.; Yamagata H. Vibronic Coupling in J-Aggregates and beyond: A Direct Means of Determining the Exciton Coherence Length from the Photoluminescence Spectrum. Journal of Physical Chemistry B 2011, 115 (18), 5133–5143. 10.1021/jp104752k. 20957993

[ref36] Bialas D.; Zhong C.; Würthner F.; Spano F. C. Essential States Model for Merocyanine Dye Stacks: Bridging Electronic and Optical Absorption Properties. J. Phys. Chem. C 2019, 123 (30), 18654–18664. 10.1021/acs.jpcc.9b04430.

[ref37] Lindorfer D.; Müh F.; Renger T. Origin of Non-Conservative Circular Dichroism of the CP29 Antenna Complex of Photosystem II. Phys. Chem. Chem. Phys. 2017, 19 (11), 7524–7536. 10.1039/C6CP08778G. 28247880

