# Supplementary material for: Unusual Photophysical Properties of Porphyrin-Based Supramolecular Polymers Unveiled: The Role of Metal Ligands and Side Group Amide Connectivity
Source: J Phys Chem C Nanomater Interfaces. 2023 Nov 21;127(48):23323–31. doi: 10.1021/acs.jpcc.3c05828 (PMC10711788; doi:10.1021/acs.jpcc.3c05828)
Supplement: Supplementary file 1 — jp3c05828_si_001.pdf [file jp3c05828_si_001.pdf]

Supporting Information

Unusual Photophysical Properties of Porphyrin-Based Supramolecular Polymers Unveiled: The Role of Metal Ligands and Side Group Amide Connectivity

[1]Ioannis Touloupas, [2,3]Elisabeth Weyandt, [2,3]E. W. Meijer, [1]*Richard Hildner

[1] *Zernike Institute for Advanced Materials, University of Groningen, Nijenborgh 4, 9747 AG Groningen, The Netherlands*

[2] *Laboratory of Macromolecular and Organic Chemistry, Eindhoven University of Technology P. O. Box 513, 5600 MB Eindhoven, The Netherlands*

[3] *Institute for Complex Molecular Systems, Eindhoven University of Technology P. O. Box 513, 5600 MB Eindhoven, The Netherlands*

*Corresponding Author:

E-Mail: r.m.hildner@rug.nl

Section S1: Q-band absorption of free-base porphyrins

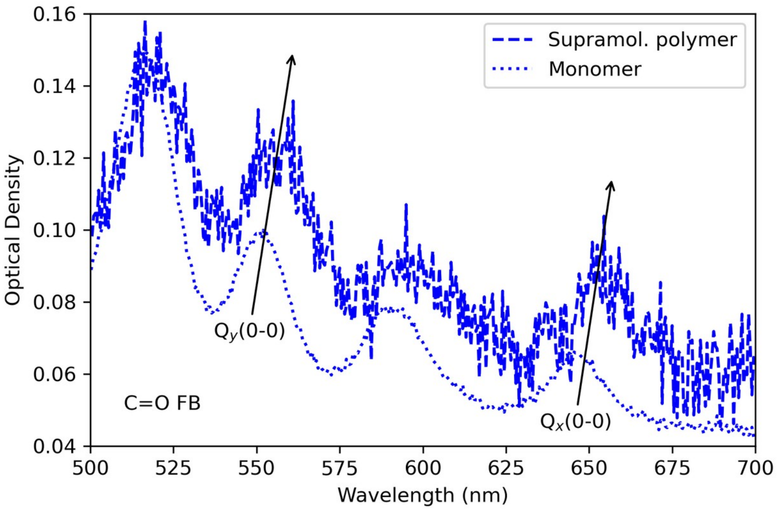

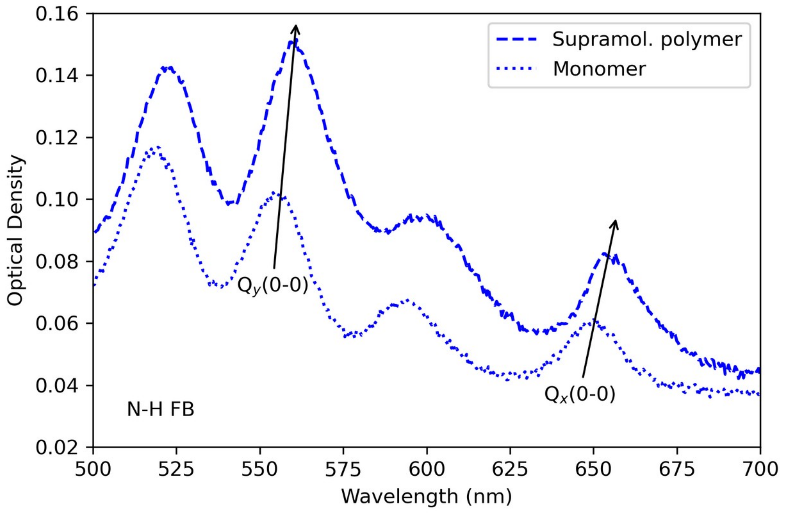

Figure S1: Q-band absorption of the free-base porphyrin derivatives with C=O centred (top) and N-H centred side groups (bottom). The dotted lines represent the absorption spectra of the molecularly dissolved monomers in chloroform, and the dashed lines depict the absorption spectra of the supramolecular polymers in MCH. The (relative) changes of the 0-0 peaks of the Q_x- and Q_y-absorptions upon supramolecular polymerisation are highlighted by arrows and are indicative of J-type aggregation with the slipped stacking of the Q-band transition dipole moments.

Section S2: Streak camera data

For all Streak camera data in this section S2 the excitation wavelength was 413 nm.

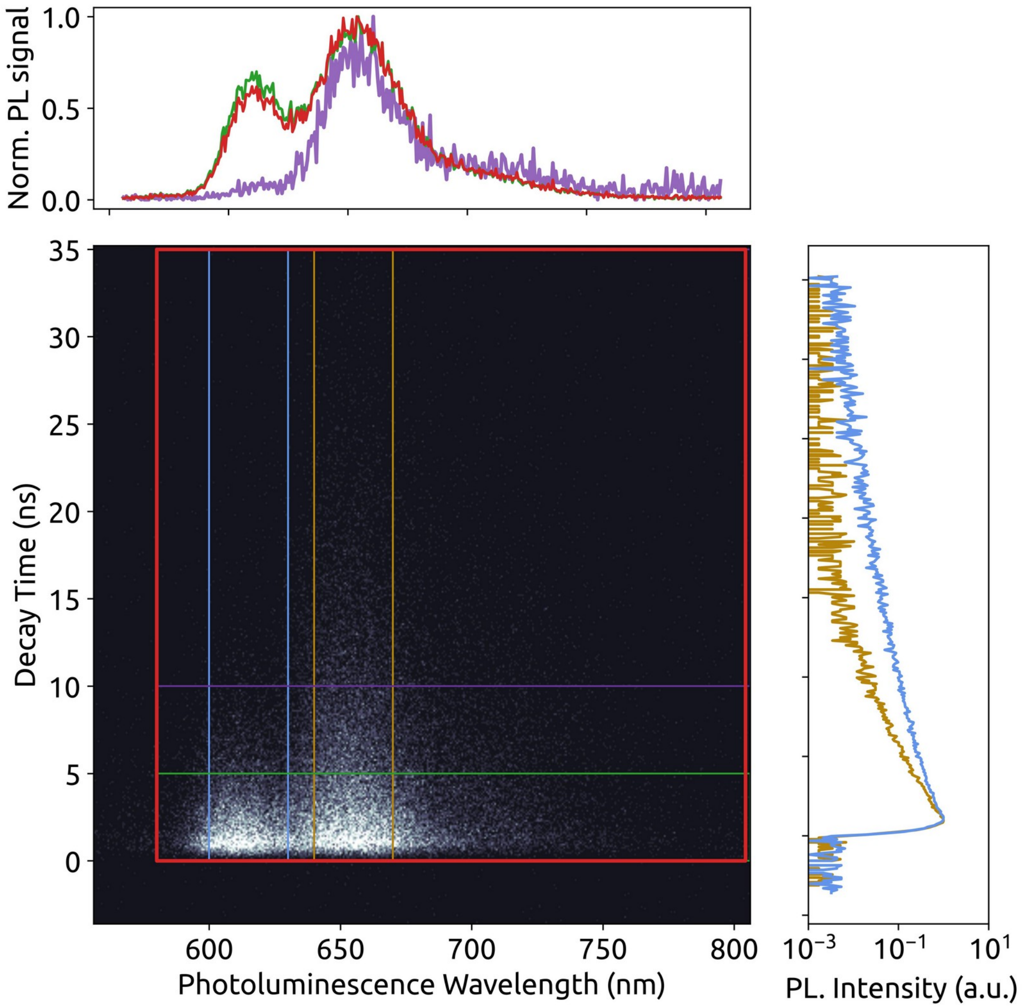

Figure S2: Streak camera data with transient spectra (top) and PL decay curves (right) for supramolecular polymers based on C=O Zn porphyrins.

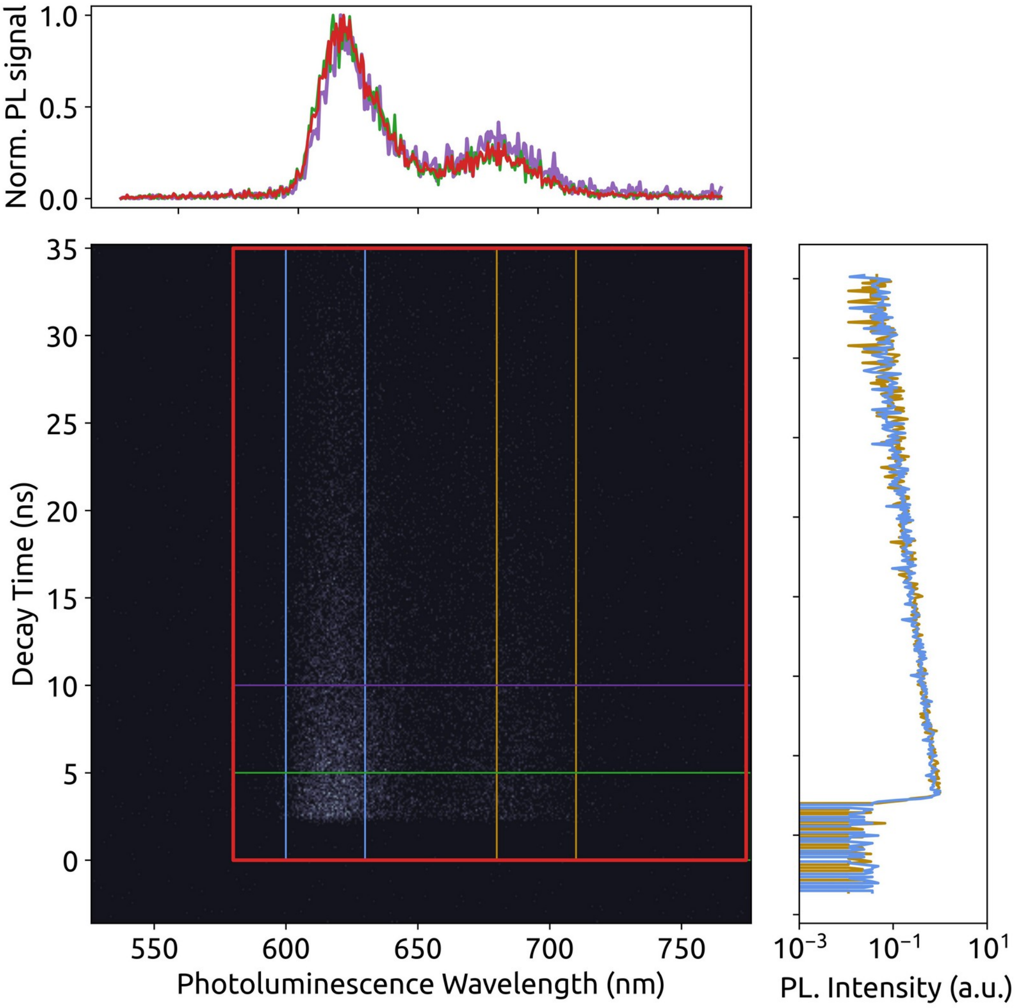

Figure S3: Streak camera data with transient spectra (top) and PL decay curves (right) for supramolecular polymers based on C=O FB porphyrins.

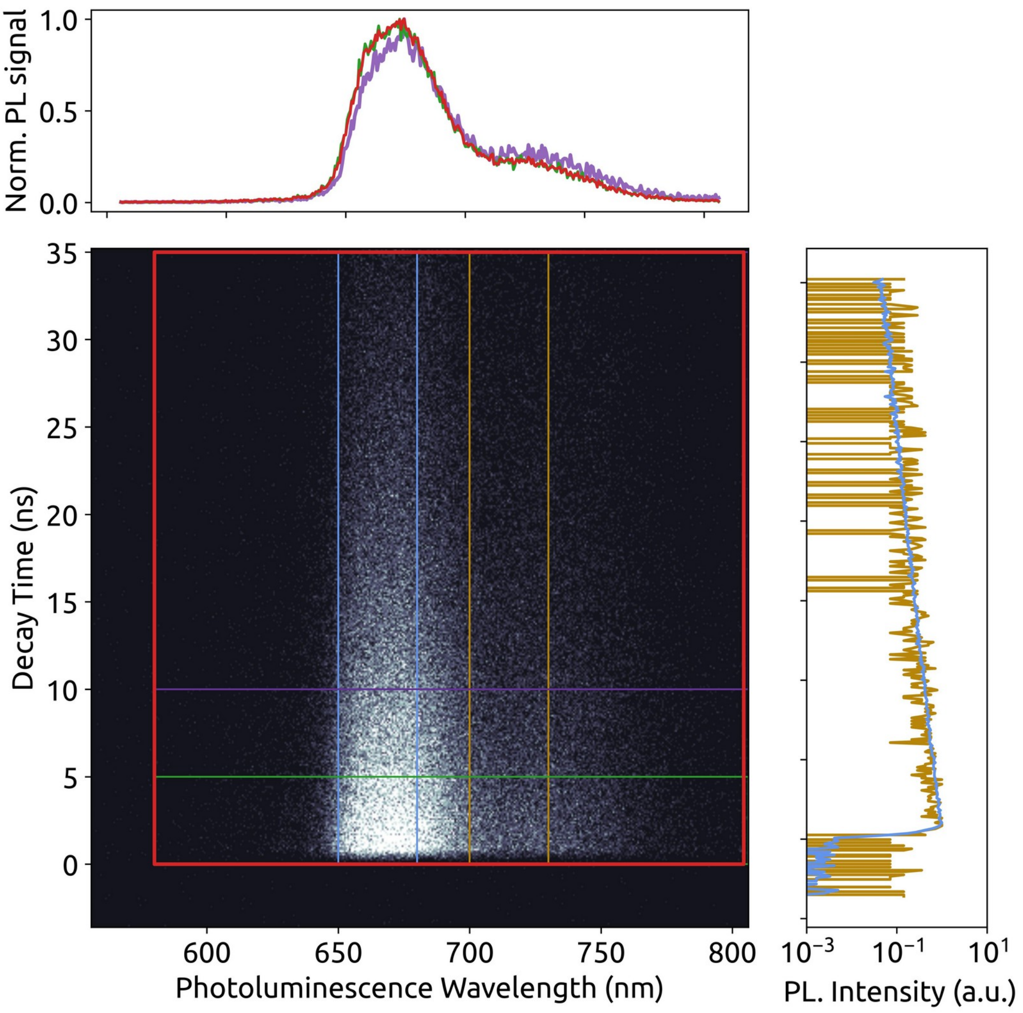

Figure S4: Streak camera data with transient spectra (top) and PL decay curves (right) for supramolecular polymers based on N-H FB porphyrins.

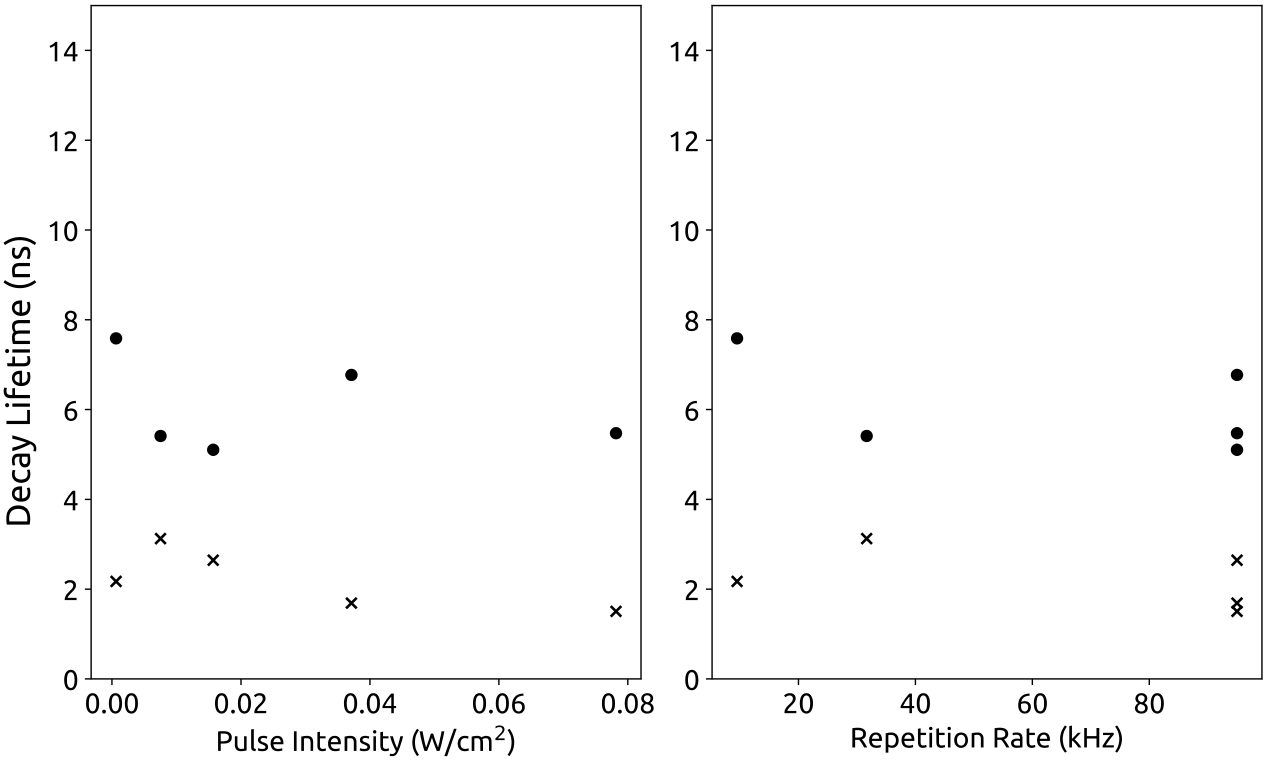

Figure S5: Lifetimes of the J_1-aggregates (dots) and J_2-aggregates (crosses) as a function of the fluence of the excitation pulses and of the repetition rate for C=O Zn based supramolecular polymers.

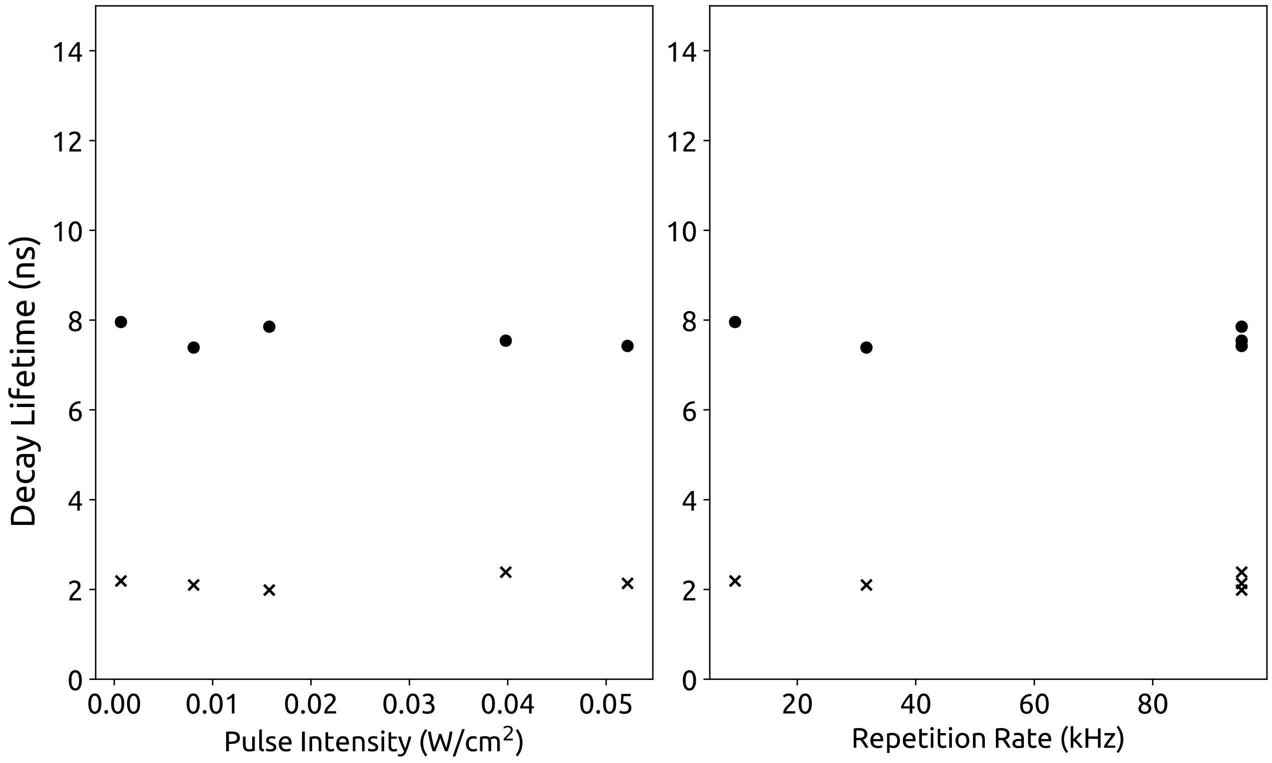

Figure S6: Lifetimes of the J_1-aggregates (dots) and J_2-aggregates (crosses) as a function of the fluence of the excitation pulses and of the repetition rate for N-H Zn based supramolecular polymers.

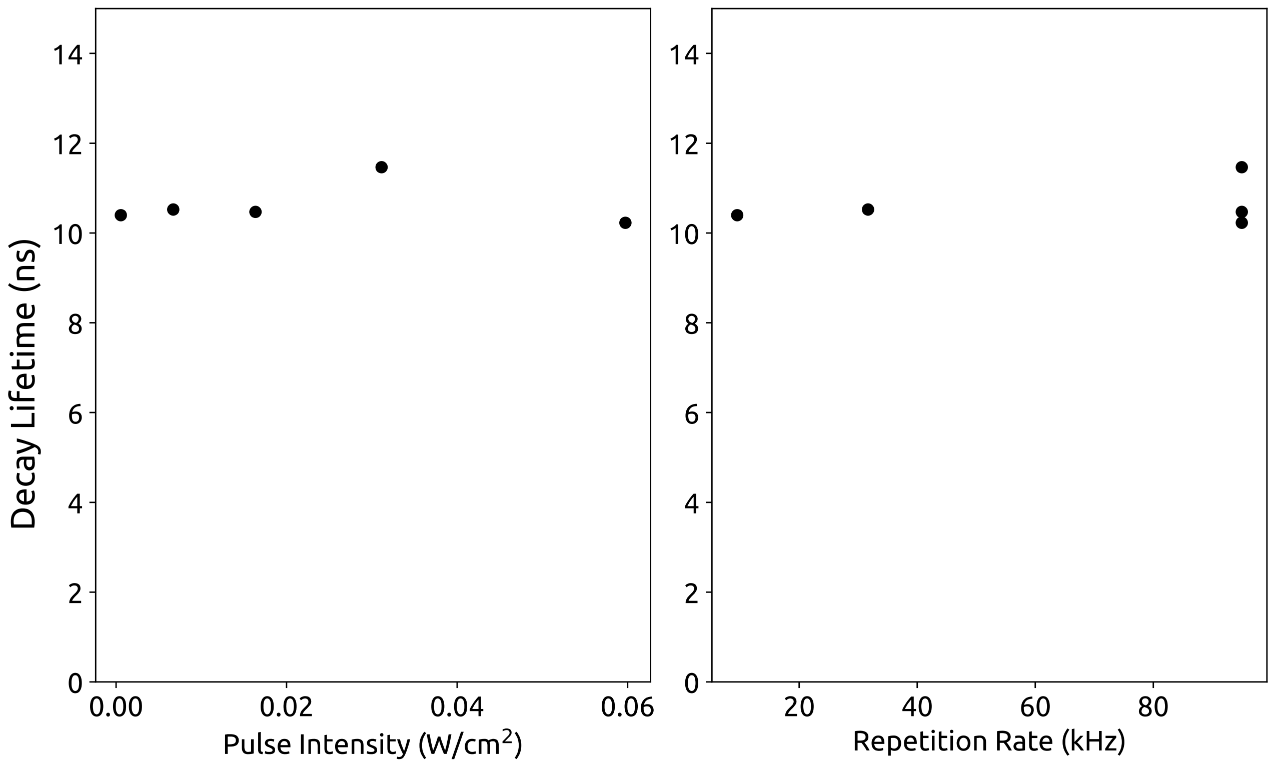

Figure S7: Lifetimes of the J-aggregates as a function of the fluence of the excitation pulses and of the repetition rate for C=O FB based supramolecular polymers.

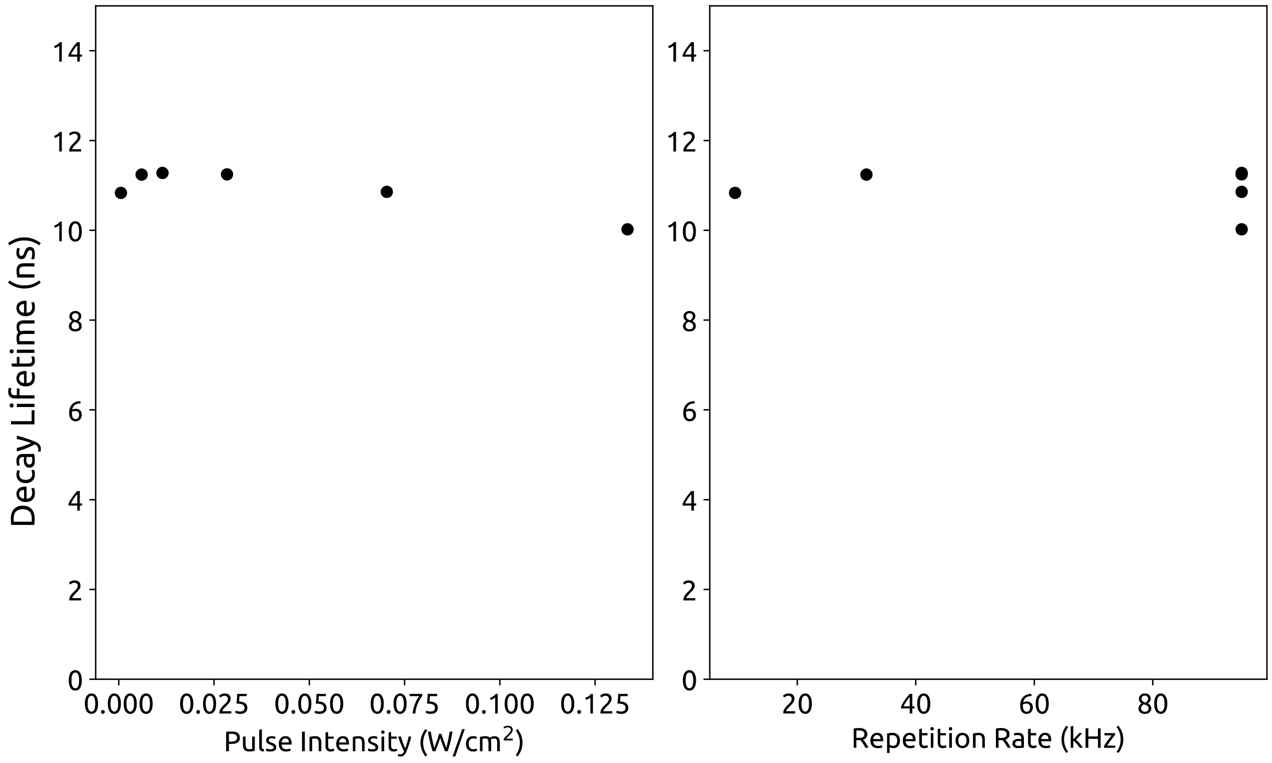

Figure S8: Lifetimes of the J-aggregates as a function of the fluence of the excitation pulses and of the repetition rate for N-H FB based supramolecular polymers.

Section S3: Non-radiative decay rates and (average) relative PL quantum yields

Since the PL quantum yield (PLQY) is very low for all compounds and close to the detection limit of our integrating sphere (~1 %), we report in Table S1 only relative PLQYs, i.e., the PLQY of the monomer relative to that of the supramolecular aggregate (see Materials and Methods section in the main text).

Table S1: Lifetimes of monomers in chloroform and aggregates in MCH, as well as the (average) relative PLQY for all porphyrin compounds studies here.

Compound	Lifetime (ns)			(avg.) rel. PLQY
	Monomer	J_1	J_2	
C=O FB	4.1	10.3	–	0.5
C=O Zn	4.2	6.2	1.9	0.2
N-H FB	4.2	10.8	–	2.3
N-H Zn	4.2	7.2	2.1	1.5

Based on the definition of the PLQY as the ratio of the radiative decay rate and the total decay rate (sum of radiative and non-radiative decay rates, which we measured, Table 1 of the main text) and the measured relative PLQY, we can calculate the ratio of non-radiative rates between aggregate and monomer $k_{nr}^{agg} \,/\, k_{nr}^{mon}$ i.e., the change of the non-radiative rate k_{nr} upon aggregation. For the free-base porphyrin aggregates, we show in Fig. S9 the ratio of non-radiative rates as a function of the PLQY of the monomers, since we do not know exactly the latter. It is clear that for realistic values of the PLQY of around (or below) 1%, the non-radiative rate in the aggregates is significantly reduced compared to that of the free-base monomers. Since for the N-H (C=O) centred free-base porphyrin aggregates the relative PLQY is increased (decreased), the ratio of the non-radiative rates show the opposite trend with increasing monomer PLQY.

For the Zn-centred porphyrin aggregates we cannot discuss such changes in rates, since we only determine average relative PLQY due to averaging over both co-existing species. It is, however, conceivable that the species with the longer lifetime and smaller linewidths (J_1) features a similar reduction in non-radiative rate. For the short lifetime species (J_2), this is less pronounced, in

agreement with the more disordered aggregates that are formed with this species (see the linewidths in Table 2 of the main text).

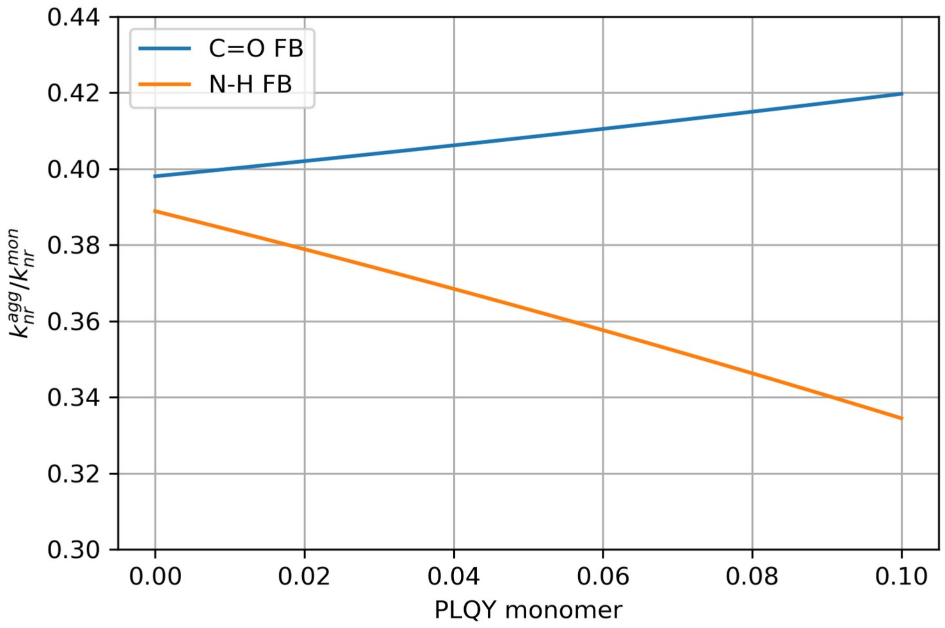

Figure S9: Ratio of non-radiative rates of aggregate and monomer as a function of the (unknown) PLQY of the corresponding monomers for the free-base porphyrins.

Section 4: Streak camera data upon excitation at 389 nm

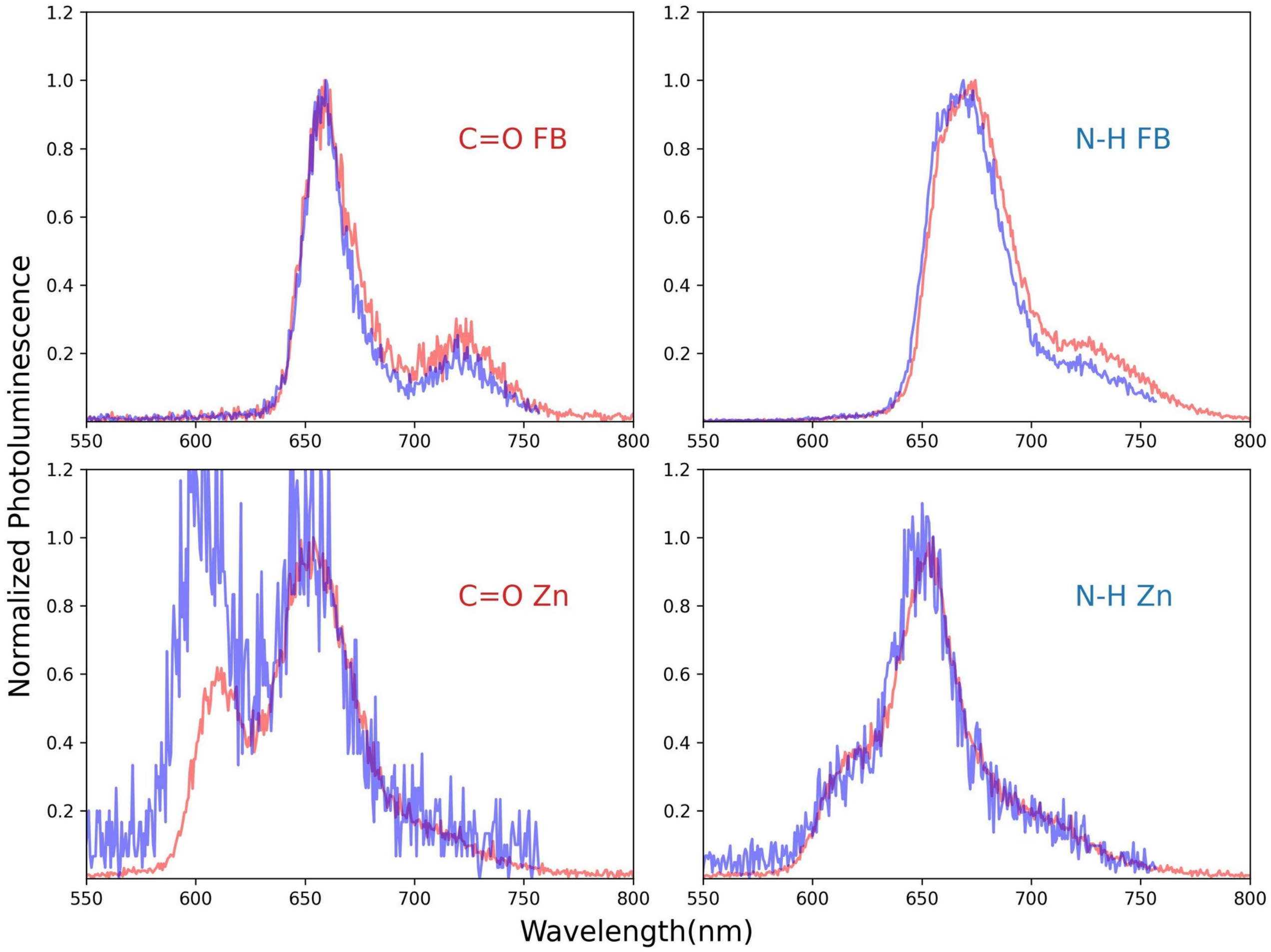

Figure S10: Time-integrated photoluminescence spectra of supramolecular polymers extracted from Streak data using excitation wavelengths of 413 nm (red) and 389 nm (blue).

To verify that the PL signal does indeed stem exclusively from supramolecular polymers and not from residual monomers in solution, we also use a different excitation wavelength of 389 nm that does not allow to excite monomers (see Fig. 2). Fig. S10 compares the time-integrated PL spectra from streak camera data for the two excitation wavelengths (blue: 389 nm; red: 413 nm) for all compounds. For all supramolecular polymers the time-integrated spectra are essentially identical, except for those based on C=O Zn. For those supramolecular polymers we observe a slightly stronger PL signal around 600 nm upon excitation at 389 nm, at which the J_2-species emits, see Fig. 4. Hence, for this system we excite this J_2-species with a higher probability at lower wavelength.

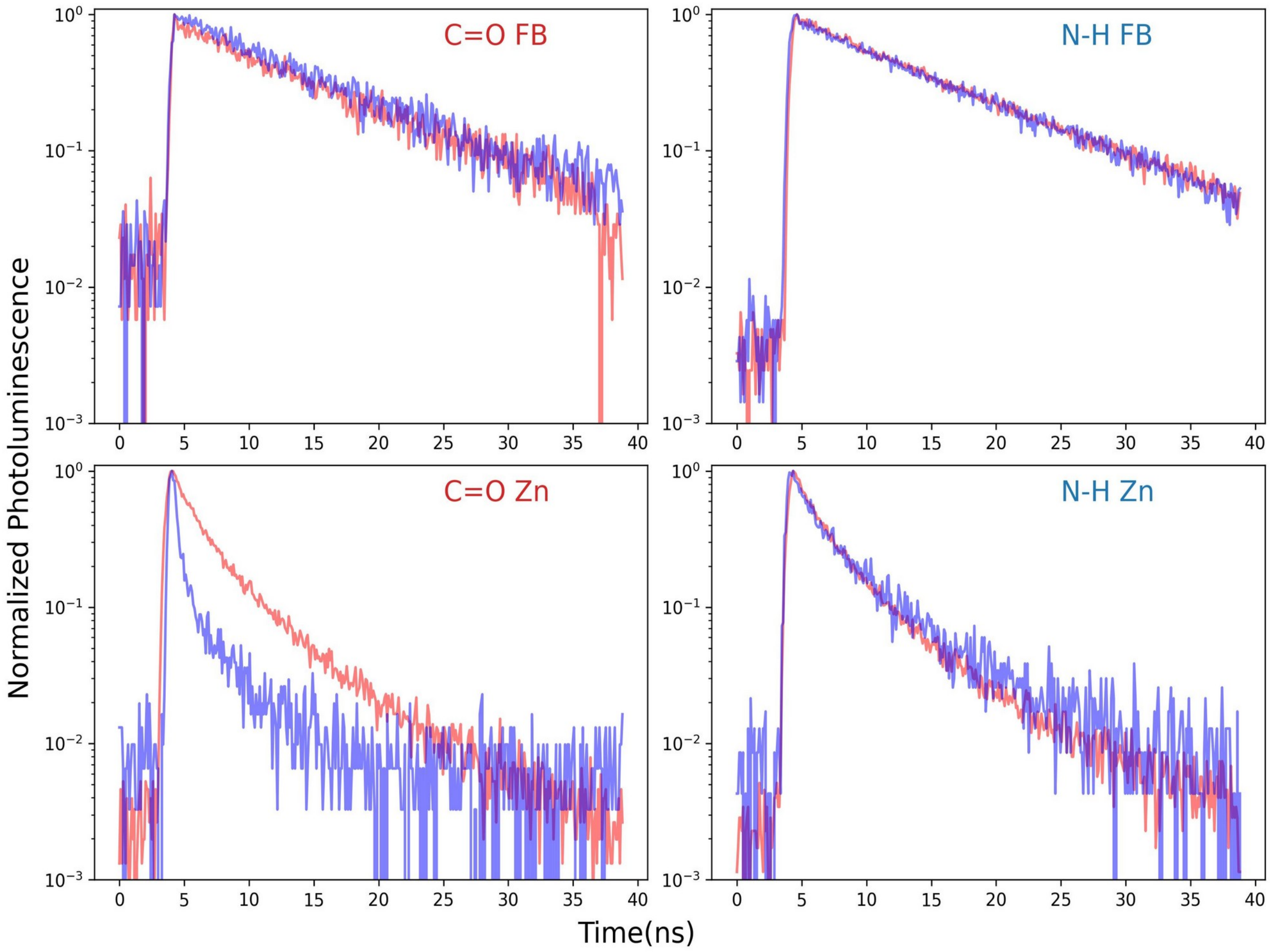

Figure S11: Spectrally integrated PL decays of supramolecular polymers extracted from Streak data using excitation wavelengths of 413 nm (red) and 389 nm (blue).

Fig. S11 compares the spectrally integrated PL decay curves (from 550 to 800 nm) of the supramolecular polymers for the two excitation wavelengths. Again we find that those are very similar, except for C=O Zn based supramolecular polymers. As outlined above, for that latter system the short-lived J_2-species (see Table 1) is excited with higher probability, and hence the decay at short times after excitation is more pronounced.

To summarise, both the spectral and lifetime data upon excitation at 389 nm and at 413 nm are consistent with PL exclusively from supramolecular polymers. There is no detectable monomer signal after self assembly. Importantly, those spectral and lifetime features are also inconsistent with null- or X-type aggregates discussed by Spano[1] and Gierschner,[2] i.e., a specific assembly of

monomers within a supramolecular polymer so that the electronic interactions cancel out (in that case no changes in lifetime and spectral shapes are observed compared to monomers).

References:

(1) Hestand, N. J.; Spano, F. C. Interference between Coulombic and CT-Mediated Couplings in Molecular Aggregates: H- to J-Aggregate Transformation in Perylene-Based π-Stacks. The Journal of Chemical Physics **2015**, *143* (24), 244707.

(2) Gierschner, J.; Shi, J.; Milián-Medina, B.; Roca-Sanjuán, D.; Varghese, S.; Park, S. Luminescence in Crystalline Organic Materials: From Molecules to Molecular Solids. Advanced Optical Materials **2021**, *9* (13), 2002251.
